# Effect of Fiber Loading on Mechanical and Flame-Retardant Properties of Poplar-Fiber-Reinforced Gypsum Composites

**DOI:** 10.3390/molecules29112674

**Published:** 2024-06-05

**Authors:** Yunpeng Ye, Qinqin Huang, Xingong Li

**Affiliations:** School of Materials Science and Engineering, Central South University of Forestry and Technology, Changsha 410004, China; 18057978983@163.com (Y.Y.); yyp20232023@163.com (Q.H.)

**Keywords:** poplar fiber, gypsum-based composites, mechanical properties, water resistance, flame retardant properties

## Abstract

Gypsum-based composites were prepared via a slurry casting process using construction gypsum as the binding material and poplar fibers as reinforcing material. The effects of different fiber content and curing time on the mechanical properties, water resistance, and flame retardancy of these composites were investigated, and the influence mechanism was characterized by infrared spectroscopy, scanning electron microscopy, and X-ray diffractometry. The results showed that the best composite mechanical strength was achieved with 10% poplar fiber- content, and the absolute dry flexural and compressive strengths reached 3.59 and 8.06 MPa, respectively. Compared with pure gypsum, the flexural strength and compressive strength increased by 10% and 19%, respectively. The inclusion of fibers somewhat prevented the migration of free water within the composites and enhanced their water resistance. At 10% fiber content, the composite’s 24 h water absorption rate was 34.3%, 8% lower than that of pure gypsum, with a softening coefficient of 0.55. However, fiber content increases the porosity of gypsum-based composites. When heated, this increased porosity accelerates’ heat conduction within the matrix, raising the peak and total exothermic rates, thereby weakening the composites’ inherently flame-retardant properties. Poplar-fiber-reinforced gypsum-based composites offered superior performance in commercial applications, compared to pure gypsum board, providing a sustainable and green alternative for ceilings, partitions, and other applications, while broadening the prospects for gypsum-based composites in the engineering field.

## 1. Introduction

Gypsum products have excellent characteristics, such as being fireproof and lightweight, which are much needed for interior and exterior wall insulation materials in the current three-step energy conservation in the construction industry [1,2]. Gypsum-based composites are used to construct walls, ceilings, and partitions, which also provide acoustic and fire protection [3,4]. However, the disadvantages of the low strength and poor water resistance of gypsum products have greatly limited their application in buildings. To overcome these drawbacks, fiber-reinforced matrix composites are usually prepared using various synthetic or natural fibers to enhance the physical and mechanical properties of the composites [5,6,7]. In the construction field, industrial fibers made of glass, basalt, polymers (polypropylene, polyester, or PVA), or steel play an important role in improving the properties of brittle matrix composites, where the addition of a large number of fibers to the matrix [8] improves the fracture toughness and resistance to crack extension and the tensile strength of brittle composites increased [9,10]. Common synthetic fibers, such as polymer, basalt, and glass fibers, have higher tensile strength than a gypsum matrix in most cases and also improve the distribution of stress loads, which helps to improve composite mechanical properties [11,12]. Fibers in the gypsum matrix in dispersed form [9,13] yield composites with better stiffness, dissipative capacity, and impact resistance, and reinforcing fibers can also absorb a large amount of energy and improve ductility [14]. However, synthetic fibers are expensive to produce and can pollute the environment during production, while natural fibers have the advantages of being lightweight, having good toughness, being low cost, they do not pollute the environment, and they have combinations of useful mechanical and physical thermal properties, which have clear advantages for manufacturing fiber-reinforced gypsum-based composites [3,15,16,17].

In recent years, there have been many attempts to study natural fibers as reinforcement for gypsum-based composites [15,16,18], and since natural fibers are environmentally friendly materials, renewable and fully recyclable, and meet the requirements of green building systems, they are commonly used to reinforce highly brittle matrices and are a major application area for construction materials [19]. Mainly including wood fibers, hemp fibers, hemp stalks, and cotton straw [20,21,22,23], natural fibers have been studied for reinforcing gypsum-based composites. Hou Zhiyi [24] et al. conducted a systematic study on the mechanical properties of gypsum-based composites with added wood fibers (1, 3, and 5 wt%), and the results showed that the addition of 3-wt% wood fibers significantly improved composite mechanical properties. Li [25] studied gypsum-based composites with raw bamboo fibers and found that the best strengthening effect was achieved at 2% content. He also found that, if the amount of admixture was too large, it was difficult to mix the fiber and composite cementing material evenly and agglomeration can occur, thus affecting composite mechanical properties. Kuqo [26] mixed wood fibers with seaweed in gypsum and found that the flexural and compressive strengths of the resulting composites increased by 28 and 4%, respectively, when the wood fiber content was 2 wt%. The above reports proved that the addition of natural fibers can improve the mechanical properties of gypsum-based composites, but most of the current studies on the dosage of natural fiber-reinforced gypsum-based composites have been controlled within 5 wt%, and there are almost no reports on the studies of high dosage of natural fiber gypsum-based composites. Due to the reason that it is difficult to mix the fibers uniformly, most scholars mainly explore the mechanism of adding a small amount of natural fibers to the compressive strength and flexural strength of gypsum-based composites, and there is no complete theoretical system for the study of highly doped natural-fiber-reinforced gypsum-based composites. The advantages of preparing highly doped natural-fiber-reinforced gypsum-based composites are: on the one hand, highly doped natural fiber can further reduce the preparation cost and density of gypsum-based composites and broaden the scope of application; on the other hand, it can be used for the full resourcefulness of some discarded natural fibers (industrial residues such as wood chips and agricultural straws, etc.).

In this study, highly doped poplar-fiber-reinforced gypsum matrix composites were prepared by adjusting the water–cement and wood–cement ratios using construction gypsum as a matrix and poplar fiber as filler. To investigate the effects of poplar fiber content on the performance and mechanism of gypsum-based composites, the optimal value of fiber content was obtained, under the premise of National Standard GB/T 17669.1-1999 [27], to increase the economic benefits of gypsum-based composites and provide technical and theoretical support for the development of gypsum-based composites.

## 2. Experimental Study

### 2.1. Experimental Raw Materials

Construction gypsum, a white powder ground from ore after dehydration, is a gelling material with calcium sulfate as the main component. The density is 2.60–2.75 g/cm^3^ and the bulk density is 800–1000 kg/m^3^. The theoretical composition of construction gypsum is CaO at 32.5% and SO_3_ at 46.6%, with some impurities, such as SiO_2_, Al_2_O_3_, Fe_2_O_3_, MgO, and SO_3_ [28], but the composition does not vary much. The gypsum used in this experiment was purchased from Jinan Riyueying Chemical Co., Ltd. (Jinan, China, 40 kg in bags) (Table 1 and Table 2).

Poplar fiber, as crushed waste poplar residue, over 10–20 mesh sieve, wood fiber length 0.5–1 cm, thickness 0.5 mm, and self-processed, was obtained from the Lumber market in Changsha City, Hunan Province. The material was dried to absolute dryness at a constant temperature of 103 ± 2 °C and stored in a sealed bag. Sodium citrate retarder, chemically pure, was purchased from Tianjin Zhiyuan Chemical Reagent Co., Ltd. (Tianjin, China).

### 2.2. Test Equipment and Instruments

A 40 × 40 × 160 mm triple test die DCS-R-100 universal mechanical testing machine (Jinan Kehui Test Equipment Co., Ltd., Jinan, China), X-ray diffractometer (XRD; Shimadzu Corp., Kyoto, Japan), MIRA3LMH type scanning electron microscope (SEM; Taseken China Ltd., Shanghai, China), and FTT007 type conical calorimeter (Combustion Technology, LLC, Vancouver, WA, USA) were used in analyses.

### 2.3. Composite Material Preparation

In this test, the fiber post-blending method was adopted, in which gypsum powder, water, wood fiber, and sodium citrate were weighed in proportion (Table 3). First, sodium citrate and water were mixed and stirred, then gypsum powder was added, with a weight ratio of water to gypsum at 0.8 wt%. Finally, poplar fiber in various proportions was well mixed in, and the resulting paste was injected into a 40 × 40 × 160 mm mold, which was then placed on a vibration table for 2 min, excess slurry scraped off the surface, and specimens de-molded after 2 h, and tested for initial strength. The rest of the specimens were tested after 7, 14, and 28 d of natural curing. All test operations and sample curing were performed indoors (~24 °C) in a ventilated, dry (~50 RH) room.

### 2.4. Performance Testing and Characterization

#### 2.4.1. Mechanical Properties Testing

The mechanical properties of poplar-fiber-reinforced gypsum matrix composites were tested using a DCS-R-100 general purpose mechanical tester (Shimadzu Co., Ltd., Kyoto, Japan) according to GB/T 17669.1-1999 “Gypsum for Construction”.

Flexural strength: The specimen was placed on its side on the two support rollers of the flexural testing machine with each edge of the specimen perpendicular to the rollers. The loading rollers were equidistant from the two supporting rollers, with a support distance of 100 cm and a test speed of 2 kN/s or 1.25 MPa/s, until the sample in the machine broke break. Three parallel specimens were tested, and the average value was taken. The flexural strength was calculated using the following formula:

*R_f_* = 1.5*F_f_* · L/b^3^

where: 

*R_f_*—flexural strength (MPa);

*F_f_*—damage load (N);

L—support cylindrical center distance (mm);

b—The side length of the square of the specimen section, 40 mm.

The calculated values are accurate to 0.01 MPa.

Compressive strength: Compressive strength was determined by cutting 6 half-pieces of specimens into 40 × 40 × 40 mm cubic specimens after performing flexural tests. During a test, the specimen was placed in the compressive jig, and the forming surface of the specimen was perpendicular to the compressive surface. The machine test speed was 2 kN/s or 1.25 MPa/s, such that the specimen was destroyed within 20–40 s after the start of loading. Five parallel specimens were tested, and the average value was taken. The compressive strength was calculated using the following formula:

*R_c_* = *P*/S

In the formula: *R_c_* is the compressive strength (MPa);

*P* is the failure load, (kN);

S is the compressive area of the specimen, 40 mm × 40 mm = 1600 mm².

The selection rules for compressive strength values are the same as those for flexural strength values, and the compressive strength of the specimen is finally calculated.

#### 2.4.2. Water Resistance Test

Water absorption test: A group of test samples was dried in an oven at 45 °C to constant weight, their mass recorded as A_1_. Then, the samples were soaked in tap water at room temperature for 2 and 24 h, and their mass was recorded as A_2_, thus obtaining the water absorption rate, expressed as (A_2_ − A_1_)/A_1_ × 100%. Three parallel samples were tested in each group and averaged.

Determination of softening coefficient: A group of samples was dried in an oven at 45 °C to constant weight and strength tests recorded as R_dry_. Another group of samples was immersed in water for 24 h and their tested strength was recorded as R_wet_, such that the softening coefficient was R_wet_/R_dry_. Three parallel samples were tested in each group and averaged.

#### 2.4.3. X-ray Diffraction Analysis (XRD)

Specimens were first sampled at different curing ages and hydration terminated with anhydrous ethanol. After 7 d, specimens were dried and ground with an agate mortar until they completely passed through a 200-mesh sieve. Tests were performed with an Empyrean X-ray diffractometer (Malvern Panalytical B.V., Almelo, The Netherlands) with a copper diffraction anode target, scanning step angle of 0.02°, and a scanning rate of 12°/min.

#### 2.4.4. Fourier Infrared-Spectroscopy Analysis (FT-IR)

A Fourier transform-infrared spectrometer (instrument model: Frontier) from PerkinElmer, Inc. (Waltham, MA, USA) was used to analyze the functional group species of poplar fiber gypsum-based composites with different content amounts. The various types of groups contained in the samples were analyzed according to the position and intensity of the absorption peaks in IR spectrograms to analyze the composition and structure of the samples by deformation. Test samples were in powder form, with a resolution at 4 cm^−1^, number of scans at 16, and data acquisition at 500–4000 cm^−1^.

#### 2.4.5. SEM Analysis

Samples were taken from the original fracture surface in the center of the specimen and soaked in anhydrous ethanol solution for 24 h to stop the hydration of the gypsum inside the sample. Subsequently, the samples were dried in a blower oven at 35 °C until the mass of the specimen was constant and then gold sputtered. SEM was used to examine the fracture surface morphology using a Hitachi Regulus 8100 instrument (Hitachi Instruments, Inc., Tokyo, Japan).

#### 2.4.6. Cone Calorimetric Analysis

An FTT0007 conical calorimeter (Fire Testing Technology, West Sussex, UK) was used, with test samples of 100 × 100 × 3 mm according to ISO 5660-1 [29]. Test specimens were placed vertically, at least 2 pieces of each specimen were prepared, the initial mass of a specimen was 30 g, the surface area 90 cm^2^, nominal duct flow rate was 24 L/s, and external radiation flow rate most commonly used at 50 kW/m^2^. The fire parameters, such as peak heat release rate (pk-HRR) and total heat release (THR) of the specimens, were tested.

## 3. Results and Discussion

### 3.1. Effects of Poplar Fiber Content on Composite Mechanical Properties

**Figure 1:**
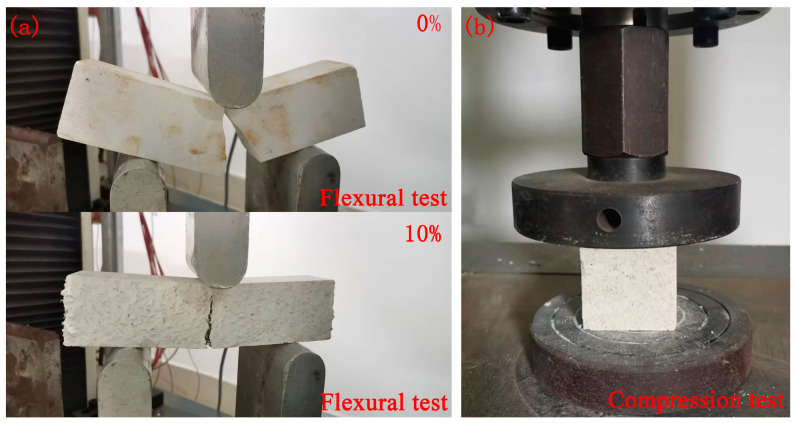
Strength test images. (**a**) Flexural test; (**b**) Compression test.

**Figure 2:**
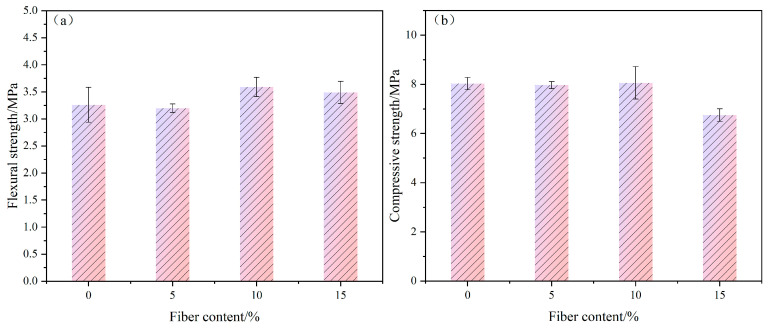
Effects of poplar fiber content on mechanical properties of composites. (**a**) flexural strength; (**b**) effect of compressive strength.

**Figure 3:**
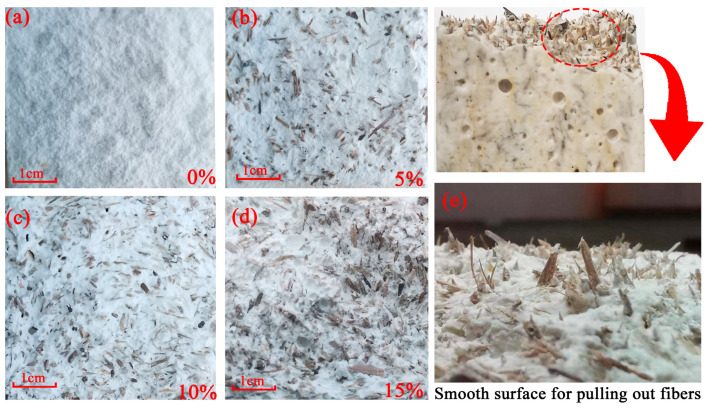
Fracture surface of gypsum-based composites with various content levels (cell phones capture optical images). (**a**) 0%; (**b**) 5%; (**c**) 10%; (**d**) 15%; (**e**) Pulled out fiber.

A histogram of the effects of poplar fiber content on the flexural and compressive strengths of gypsum-based composites showed that with increased poplar fiber content, composite flexural and compressive strengths increased and then decreased (Figure 1 and Figure 2, Table 4). Among these, at 0–5-wt% fiber content, composite integrated strength all decreased, which might have been due to the fact that the content amount was low and fiber spacing too large to transfer loads to each other (Figure 3b). The addition of poplar fibers destroyed the crystal lap structure inside the gypsum matrix, thus reducing gypsum strength. Load transfer between poplar fibers and gypsum was mainly through interfacial adhesion and mechanical engagement forces, but adhesion between poplar fibers and gypsum was limited with low fiber content. When the ends of the composite were stressed, the gypsum matrix fractured and poplar fibers pulled out directly from the gypsum matrix (Figure 3e), and thus could not play a flexural load-bearing role and reduced specimen flexural and compressive strengths [30]. The comprehensive strength of specimens all increased and then decreased when the amount of poplar fiber content was from 5 to 15 wt%, where the flexural and compressive strengths of the specimens reached the highest values of 3.59 and 8.06 MPa, respectively, at 10 wt% content. The flexural strength increased by 10% compared to pure gypsum, because when the amount of poplar fiber content reached a critical point, fibers were able to partially inhibit crack expansion during the process of pulling out, although fibers were not well bonded to the matrix material. The distance between the fibers was close and evenly distributed, such that an applied load was transferred to the fibers and distributed to the matrix between the fibers, thus reducing the chance of stress concentration to a greater extent (Figure 3c). According to the fiber crack-blocking effect [31], the dispersion of fibers within the gypsum base facilitates the crack resistance and strength of the specimens. However, When the amount of poplar fiber content exceeded 10 wt%, the gypsum matrix could not completely cover the fibers, such that fibers agglomerated, and stress concentration points appeared inside the composite (Figure 3d). Poplar fiber is a hydrophilic material, which very easily absorbs water and will affect the gypsum hydration system and composite strength.

### 3.2. Effects of Curing Time on Composite Physical and Mechanical Properties

**Figure 4:**
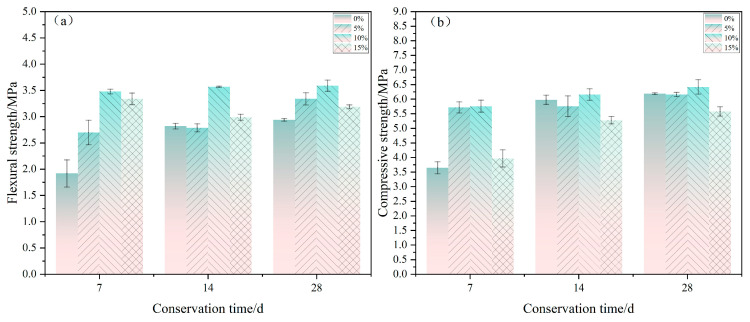
Effects of poplar fiber content on the mechanical properties of composites at different curing times. (**a**) flexural strength; (**b**) compressive strength.

The flexural and compressive strengths of gypsum-based composites increased with increased curing time, and the curing time had a common effect on the growth of flexural and compressive strengths of these composites (Figure 4 and Table 5). The reason for this effect was that the incorporation of poplar fibers hindered the hydration reaction of gypsum within the composite, but the hydration of gypsum in the composite became more complete with longer curing time. The hydration reaction of gypsum produces more water-hardening hydration products. These products allow the gypsum and wood fibers in the composite to be more tightly bound together. In the later stage of curing, the increased strength of gypsum-based composites mainly depended on water evaporation, such that the longer the curing time was, the less water there was in the material. Then, the more stable the crystalline contact points in the crystallizing process were, the less prone to distortion and deformation and less prone to dissolution of crystal contact points in a dry environment, as dissolution leads to reduced structural strength.

### 3.3. Water Resistance Test of Poplar Fiber Content on Composites

**Figure 5:**
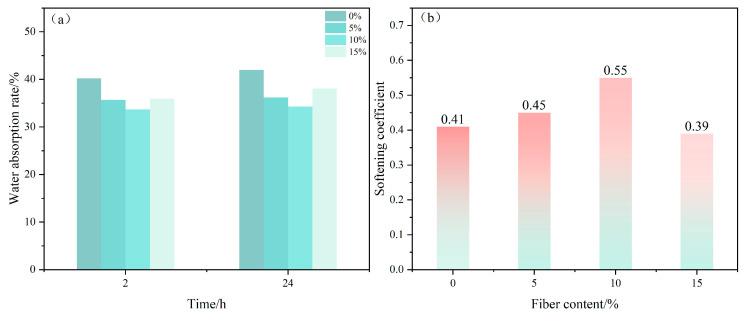
Effects of poplar fiber content on water resistance of composites. (**a**) Water absorption rate; (**b**) Softening factor.

A histogram of the effects of poplar fiber on the water absorption and softening coefficient of gypsum-based composites at 2 and 24 h showed that the trend of water absorption decreased and then increased (Figure 5). In contrast, the softening coefficient first decreased, then increased, and decreased again. This was because, to ensure the fluidity of gypsum slurries, the slurry mixing process often added much more mixing water (70%) than the theoretical water requirement, and the excess water later evaporated to form many holes after the gypsum hydrated and hardened. These holes easily formed water seepage channels, allowing the gypsum matrix to easily absorb water. Gypsum-based composites with 10-wt% poplar fiber reached a minimum water absorption of 34% and a corresponding maximum softening coefficient of 0.55. The composite material with added fibers has lower water absorption and higher water resistance, with some researchers reaching the same conclusion [32]. The reason for this was that, when the composites were exposed to water, fibers inside the gypsum base effectively absorbed the surrounding water, reduced the chance of water contact with CaSO_4_·2H_2_O crystals, reduced water dissolution on gypsum hydration products, and thus improved composite water resistance.

### 3.4. XRD Analysis of Poplar-Fiber-Reinforced Gypsum-Based Composites

**Figure 6:**
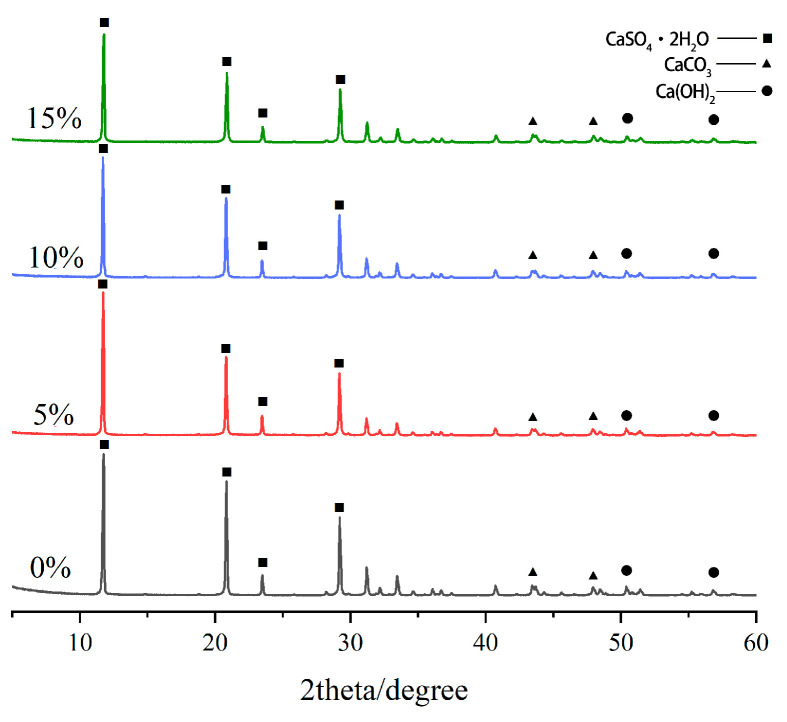
XRD diffraction patterns of composites with different poplar fiber content.

XRD diffractograms of the internal hydration products of these composites with different amounts of fiber content showed that the fiber content amount had little effect on crystals in composites in the test range (Figure 6). The diffraction peaks of CaSO_4_·2H_2_O in gypsum crystals were mainly concentrated at 11.5, 21, 23, and 29°, and those of Ca(OH)_2_ were mainly concentrated at 51 and 56°, and some diffraction peaks of the impurity CaCO_3_ were also found at 44 and 48° [33], and no diffraction peaks of new substances were detected.

The gypsum hydration reaction equation is CaSO_4_· $\frac{1}{2}$ H_2_O + $\frac{3}{2}$ H_2_O → CaSO_4_·2H_2_O. As hydration proceeded, the amount of gypsum dihydrate crystals increased, water gradually decreased, and friction and bonding between crystals increased. Structural strength thus increased, crystals gradually grew and interlaced, and the slurry gradually developed strength and grew until completely dry. In comparison with the internal hydration products of pure gypsum, it was found that the diffraction peaks (11.5, 21, and 31°) of the gypsum-based composites with the addition of poplar fibers were shorter. This implied that there was less gypsum dihydrate phase in this direction and no diffraction peaks of other new substances appeared. Meanwhile, the diffraction peaks of pure gypsum were sharper, indicating that the addition of wood fibers had a barrier effect on the crystallization of the hydration products of gypsum and that the content was reduced. The degree of gypsum crystallization in this direction was weakened, and gypsum crystallinity and cell size changed [34]. The mechanical strength of these composites was related to the number and size of gypsum crystals, and the general weakening of crystallinity implied a looser crystal lap structure and decrease in strength. This also explained the reduced strength of the composites with small amounts of added poplar fibers from a microscopic point of view.

### 3.5. FT-IR Analysis of Poplar-Fiber-Reinforced Gypsum-Based Composites

**Figure 7:**
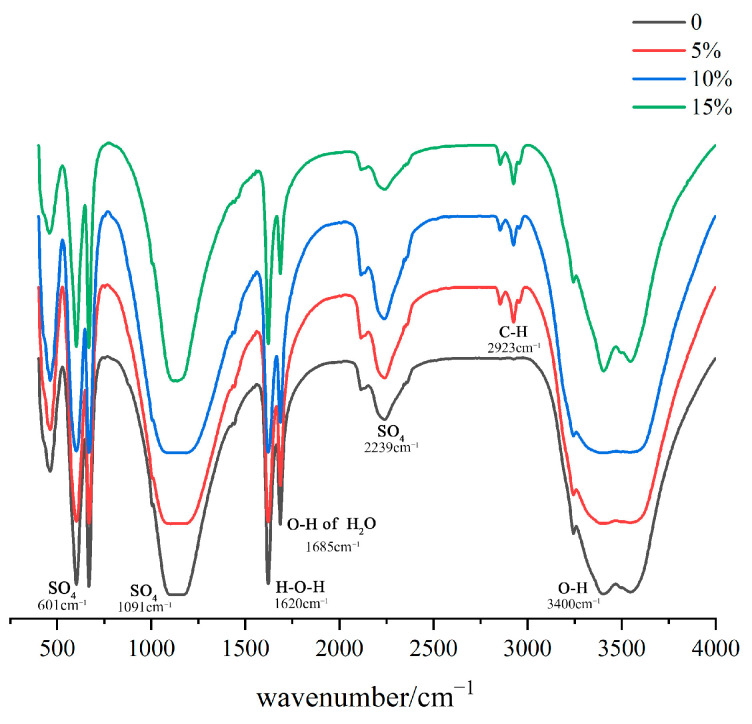
FT-IR images of poplar fiber composites with different content levels.

The bond energy changes of the gypsum-based composites with the addition of poplar fibers showed that the absorption peaks at 1685 and 3400 cm^−1^ belonged to the H_2_O-bending vibrations in the gypsum crystal hydrate and O–H-stretching vibration of the hydroxyl group of poplar fibers, respectively (Figure 7). The peak at 2923 cm^−1^ corresponded to characteristic stretching vibrations of the C–H bond of the hydrocarbon side chain in poplar fibers. The peaks at 601, 1091, and 2239 cm^−1^ were characteristic peaks of SO_4_^−^ in gypsum. The absorption peak at 3400 cm^−1^ became wider when the fiber content amount was 5 and 10%, because hydrogen (H-)-bonding weakened electron clouds and chemical bonds. Thus, the chemical bonding force decreased, and vibration frequency became smaller, with H-bonds becoming fragile, such that chemical bonding characteristic frequencies were diversified and absorption peaks became wider. The sharpening of the O–H characteristic peak at 15-wt% content indicated that, as the poplar fiber content increased, hydroxyl groups inside the composites also increased. This indicated that, from the microscopic point of view, poplar addition did not generate new chemical groups, and there were no chemical reactions between them.

### 3.6. SEM Analysis of Poplar-Fiber-Reinforced Composites

**Figure 8:**
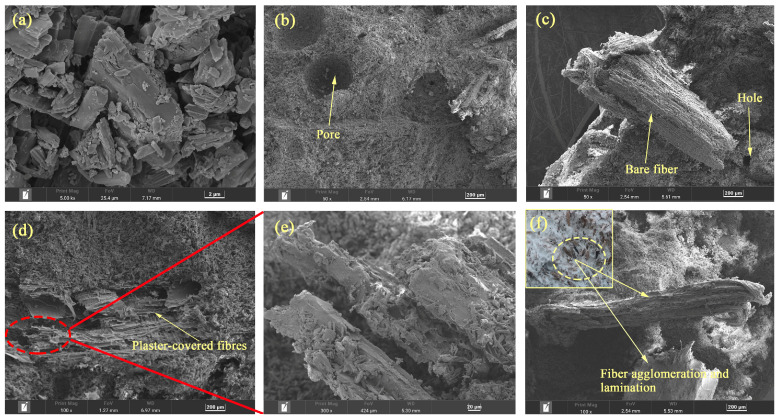
Microscopic morphology of composite cross-sections with different fiber content. (**a**) Gypsum Crystals; (**b**,**c**) 5 wt%; (**d**,**e**) 10 wt%; (**f**) 15 wt%.

The fracture surface of the pure gypsum-based paste was observed and analyzed by SEM. There were some differences observed in microstructures due to different fiber content. Pure gypsum without added fiber was characterized by a homogeneous microstructure with visible columns of gypsum crystals of similar composition and size, with gypsum crystals characterized by hexagonal faces and plate-like shapes (Figure 8a). An SEM image of 5-wt% content showed that less gypsum was connected to the matrix on exposed fiber surfaces, mainly because of their morphological characteristics and low bonding (Figure 8b,c). When the length of poplar fibers was small and the content amount low, fibers were not fully distributed within the gypsum matrix, and the fiber spacing was large. These fibers could not play the role of bridges for transferring load and had little restraining effects on cracks, and thus a strengthening effect on the gypsum matrix was not clear. Additionally, fiber addition blo cked gypsum crystals from lapping each other, resulting in a large number of voids and pores in the gypsum matrix, thus affecting composite mechanical properties. SEM images of 10-wt% content showed that gypsum was uniformly attached to poplar fibers, and the fibers tightly bonded to gypsum with no clear gaps between them (Figure 8d,e). The fibers were uniformly distributed in the gypsum and their anisotropic distribution transferred the load-bearing load to other parts of the structure, which contributed to improving composite mechanical properties. SEM images of 15 wt% content showed clumping and bunching of fibers, which was due to the fact that excess fibers led to reduced fiber binding to the matrix (Figure 8f). According to the fiber spacing theory proposed by Romualdi [35], when stress concentrations occur and develop into microcracks, the cracks are restricted by fibers during expansion, leaving only tiny and disconnected pores, and, when the fiber content increases within a certain range, the strengthening effect of the fibers diminishes. Poplar fibers have a certain width and, with the distance between poplar fibers close to each other, there can be lamination, with no matrix filling the fiber lamination, resulting in local defects. This results in a change in porosity within the composite, and the change in porosity also results in the mechanical properties of the specimen being adversely affected [36,37], leading to a reduction in strength, and therefore further addition of fibers will deteriorate the mechanical properties to a large extent.

### 3.7. Effects of Poplar Fiber Content on Flame-Retardant and Smoke Suppression Properties of Composites

#### 3.7.1. HRR and THR Analysis

HRR refers to the heat released when the material burns per unit time and is one of the most important parameters when a material burns, reflecting how fast or slow the material releases heat as it burns. Combining HRR and THR can evaluate the combustibility and flame retardancy of materials as a more objective and comprehensive analysis of material fire performance.

**Figure 9:**
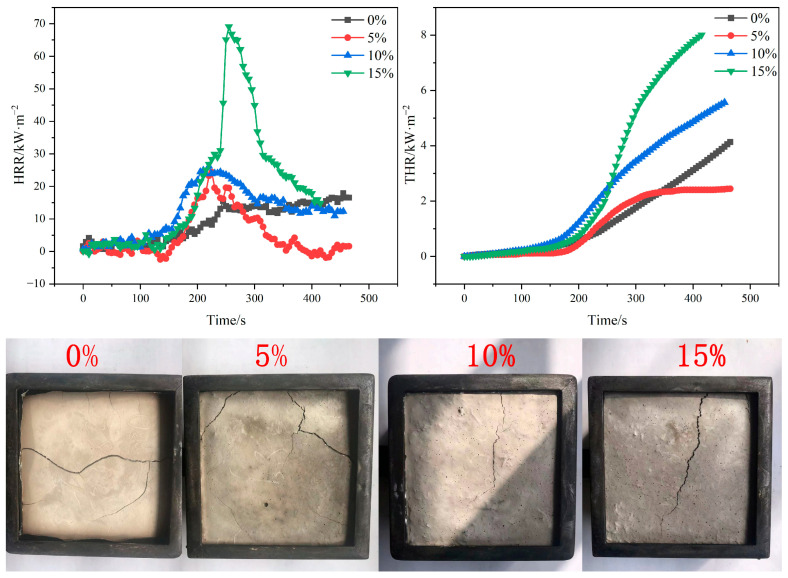
HRR and THR of composites doped with different poplar fibers and residual carbon images after flame-retardant testing.

The diagram shows the HRR and THR curves of gypsum-based composites with different poplar fiber contents (Figure 9). As seen in the figure, pure gypsum has the lowest peak (pk-HRR). The HRR of the composites gradually increased with increasing poplar fiber content. The HRR values of the composites were highest at 284 s when the poplar fiber content was 15 wt%. This is due to the fact that wood fibers are flammable, have a high calorific value, and release a large amount of heat when burned. So, the higher the fiber content in the composite material, the higher its HRR.

The THR curves of composites with different poplar fiber contents are shown. After the samples were exposed to thermal radiation for 200 s, the THR curves of samples with poplar fiber content of 15 wt% showed the greatest rate of increase. This indicates that the samples with high fiber doping burned more vigorously. The reason for this may be related to the internal water of crystallization content of the material. CaSO_4_· $\frac{1}{2}$ H_2_O reacts sufficiently with water to produce CaSO_4_·2H_2_O, in which the water of crystallization [38] in CaSO_4_·2H_2_O not only helps the bonding between the composites, but also acts as a flame retardant when the material encounters high temperatures. However, when poplar fibers are incorporated into the gypsum matrix, some of the free water required for the hydration reaction of gypsum will be absorbed by the poplar fibers, resulting in a decrease in the water of crystallization generated by hydration and a deterioration in the fire resistance of the material.

#### 3.7.2. Smoke Generation Rate and Total Smoke Generation

The smoke generation rate (SPR) is the ratio of sample-specific extinction area to sample mass loss rate. Specific extinction area indicates the smoke produced by volatilizing a unit mass of material. The total smoke generation rate (TSR) indicates the total amount of cumulative smoke generated during combustion per unit sample area, which can be calculated from the SPR integral. Combustion is a rapid oxidation process in which not only is a large amount of heat radiated, but a large amount of smoke and gases is also generated, both of which are toxic to living things. These smoke and toxic gases might be far more threatening to the lives of personnel than flame and heat.

**Figure 10:**
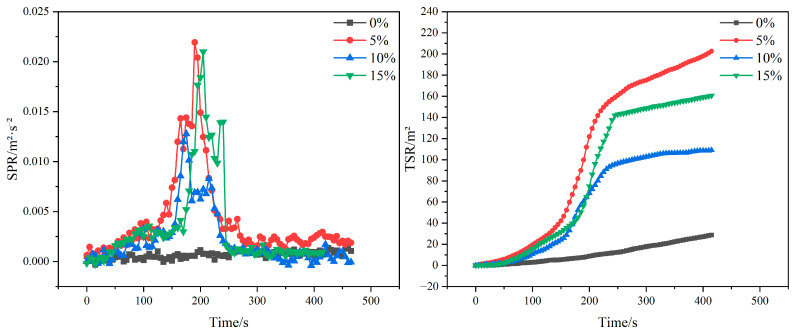
SPR and TSR of composites with different poplar fiber content.

The SPR and TSR curves of gypsum matrix composites doped with different contents of poplar fibers are shown (Figure 10). As can be seen in the figure, pure gypsum is virtually incombustible. With the addition of poplar fibers, the SPR and TSR values of the composites increased significantly, reducing the flame retardancy of the materials. This is due to the fact that the water of crystallization in gypsum determines the fire resistance of the material, and the greater the poplar fiber/gypsum mass ratio in the composite, the smaller the percent of gypsum content, and the worse the relative material fire resistance.

Comparing the SPR and TSR curves of gypsum-based composites with three different poplar fiber additions, it can be seen that the SPR and TSR peaks of the composites, when doped with 10 wt% poplar fibers, are the smallest among all the dosages. This may be due to the dispersion of poplar fibers within the matrix. When poplar fibers are uniformly dispersed in the matrix, a cohesive phase flame-retardant effect is formed, which results in the formation of a charred layer after combustion. This carbonized layer somewhat isolates the exchange of heat and oxygen, resulting in incomplete combustion of the material and reduced smoke emissions.

#### 3.7.3. Carbon Monoxide Release Rate (COP) and Carbon Dioxide Release Rate (CO_2_P)

Carbon monoxide and carbon dioxide (CO and CO_2_, respectively) are the main toxic gases released from the combustion of recombinant bamboo in fires, and the asphyxiating effect of combustion gases can be a great obstacle to evacuation and rescue activities [39]. Therefore, the study of the CO and CO_2_ release rates (COP and CO_2_P, respectively) of material combustion in fires is essential in reducing casualties.

**Figure 11:**
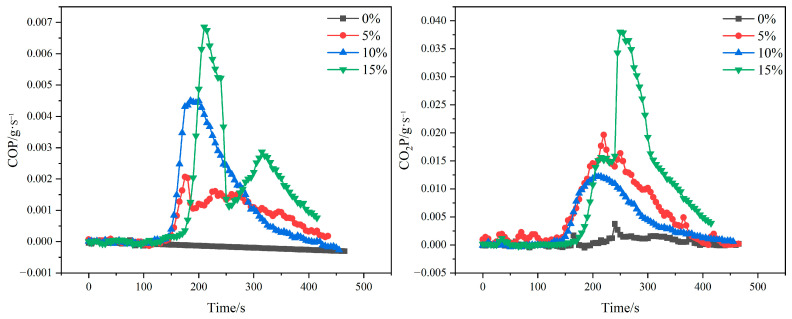
COP and CO_2_P of different poplar-fiber-doped composites.

The curves of CO and CO_2_ release rate with time for gypsum matrix composites with different poplar fiber contents are shown (Figure 11). As can be seen in the figure, the CO_2_P curve of the specimen is more similar to the change rule of its HRR curve. This indicates that the heat release of the composite specimen at high temperature is mainly provided by the reaction of CO_2_ generation [40]. The COP and CO_2_P release rates of the specimens gradually increased with the addition of poplar fiber. The peaks of COP and CO_2_P of the specimens were highest when the poplar fiber content was 15 wt%. This is due to the fact that poplar fibers release large amounts of CO and CO_2_ when burned. It can be seen that the higher the wood fiber content, the higher the CO and CO_2_ emissions of the composite material.

From the peak COP and CO_2_P curves, it can be seen that with the increase of poplar fiber content, the time taken by the composites to reach the peak COP and CO_2_P increases gradually, and at the same time, the composites’ COP reaches the peak earlier than CO_2_P reaches the peak. This suggests that the addition of poplar fibers retards the rate of CO and CO_2_ production during combustion of the composite. It was also found that the composites mainly release CO gas during the initial combustion phase. The main reason for this phenomenon is that poplar fibers are partially charred during the initial combustion process, which makes the surface layer of the material generate a charred layer with thermal insulation and oxygen barrier, under whose protection the internal material is mainly subjected to incomplete combustion. At the same time, gypsum in the combustion process, gypsum crystals in the crystalline water under the action of heat to form water vapor reduces the oxygen concentration, so that the incomplete combustion of the material phenomenon intensified. Therefore, the pre-composite material did not generate a large amount of CO_2_, but was mainly released as CO.

## 4. Conclusions

The aim of this paper is to investigate the effect of poplar fiber addition on the properties of gypsum-based composites. The main conclusions of this work can be drawn as follows:

1. The mechanical strength of gypsum-based composites increased and then decreased with increased poplar fiber content, with the mechanical strength and water resistance of the material reaching a peak with 10-wt% fiber content Among the examined composites, the 2 h flexural and compressive strengths were 2.1 and 3.48 MPa, respectively, reaching a 2.0 grade in the scale of GB/T 9776-2008 building plaster.
2. The combination of poplar fibers and gypsum base was only a physical bonding, with no chemical reactions and no new material produced.
3. The addition of poplar fibers reduced composite brittleness and enhanced crack resistance. Although the poplar-fiber-reinforced gypsum-based composites exhibited improved mechanical properties, it was not much.
4. The bonding state of poplar fiber and gypsum surface shows a positive correlation with the mechanical strength, and the closer the internal structure of the composite material is connected, the more obvious its mechanical properties will be improved.
5. The pk-HRR and THR of gypsum-based composites gradually increased with increased fiber. The appropriate amount of fiber formed a good cohesive phase with the gypsum matrix to play a flame-retardant effect and reduce TSR.
6. Partial charring of poplar fibers during combustion forms a carbonized layer, which slows down the release of CO and CO_2_ from the composite material, and at the same time, the crystalline water in the gypsum crystals forms water vapor under the action of heat and reduces the oxygen concentration, which leads to the intensification of incomplete combustion of the composite material.

Limitations and future perspectives of this study: Only the properties of a natural fiber-reinforced gypsum-based composite were explored; the length and diameter of the natural fibers also have an important effect on the mechanical strength of gypsum-based composites, which need to be further investigated in our subsequent work. By improving the treatment process of the fibers and the preparation method of the composites, the strength, toughness, and durability of the material can be further improved, the quality of the product can be enhanced, and the application and promotion of this material in the construction field can be promoted.

## Figures and Tables

**Table 1:** Main chemical composition of construction gypsum for this test (mass fraction wt%).

Ingredients	SiO_2_	Fe_2_O_3_	Al_2_O_3_	MgO	SO_3_	CaO
Content	2.74	0.40	0.99	0.80	43.08	37.44

**Table 2:** Technical specifications of construction gypsum for this test.

Technical Specifications	2 h Flexural Strength (MPa)	2 h Compressive Strength (MPa)	Fineness (0.2 mm Square Hole Sieve Residue, %)	Initial Setting Time (min)	Final Setting Time (min)
Indicator value	2.11	3.9	10.0	6	30

**Table 3:** Test principle proportioning.

Test Number	Poplar Fiber, g	Water, g	Gypsum, g	Sodium Citrate, g	Mesh
1	0	3000	3750	1.88	10–20
2	187 (5-wt% gypsum)	3000	3750	1.88	10–20
3	375 (5-wt% gypsum)	3000	3750	1.88	10–20
4	562 (15-wt% gypsum)	3000	3750	1.88	10–20

**Table 4:** Error table of the effect of wood fiber addition on composite mechanics.

Fiber Dose (wt%)	Flexural Strength (MPa)	Compressive Strength (MPa)
0	3.26 ± 0.32	8.03 ± 0.25
5	3.2 ± 0.07	7.97 ± 0.14
10	3.59 ± 0.17	8.06 ± 0.66
15	3.49 ± 0.20	6.75 ± 0.25

**Table 5:** Error table of the effect of maintenance time on the mechanics of composite materials.

Fiber Dose	7 d Strength/MPa		14 d Strength/MPa		28 d Strength/MPa	
	Flexural Strength	Compressive Strength	Flexural Strength	Compressive Strength	Flexural Strength	Compressive Strength
0	1.92 ± 0.2	3.65 ± 0.2	2.82 ± 0.05	5.98 ± 0.15	2.94 ± 0.03	6.19 ± 0.03
5	2.7 ± 0.23	5.72 ± 0.18	2.79 ± 0.07	5.76 ± 0.3	3.34 ± 0.11	6.15 ± 0.06
10	3.48 ± 0.04	5.76 ± 0.2	3.57 ± 0.01	6.16 ± 0.19	3.59 ± 0.1	6.42 ± 0.24
15	2.97 ± 0.11	5.46 ± 0.18	2.99 ± 0.05	5.28 ± 0.12	3.19 ± 0.03	5.58 ± 0.15

## Data Availability

Data are contained within the article.
