# Correction: Ye et al. Effect of Fiber Loading on Mechanical and Flame-Retardant Properties of Poplar-Fiber-Reinforced Gypsum Composites. *Molecules* 2024, *29*, 2674

**DOI:** 10.3390/molecules30214162

**Published:** 2025-10-23

**Authors:** Yunpeng Ye, Qinqin Huang, Xingong Li

**Affiliations:** School of Materials Science and Engineering, Central South University of Forestry and Technology, Changsha 410004, China; 18057978983@163.com (Y.Y.); yyp20232023@163.com (Q.H.)

## Equal Contribution

In the original publication [1], the authors Yunpeng Ye and Qinqin Huang’s equal contribution were not included. The inclusion of this note would ensure appropriate recognition of all authors’ contributions, which is essential for maintaining transparency and scientific integrity.

The authors state that the scientific conclusions are unaffected. This correction was approved by the Academic Editor. The original publication has also been updated.
